# Hypercapnia Effects on Lung Function

**DOI:** 10.1002/cph4.70104

**Published:** 2026-01-23

**Authors:** Seungseo Choi, Laura A. Dada, Jacob I. Sznajder

**Affiliations:** ^1^ Division of Pulmonary and Critical Care Medicine, Feinberg School of Medicine Northwestern University Chicago Illinois USA

## Abstract

The biological effects of hypercapnia on the lungs have been controversial. Earlier publications suggesting that hypercapnia was beneficial for mechanically ventilated patients with acute lung injury led to the clinical paradigm of “permissive hypercapnia”. However, more recent studies have challenged this paradigm by reporting that hypercapnia activates signaling pathways with deleterious effects. This review focuses on the effects of elevated CO_2_ as a signaling molecule, highlighting the pathways and processes contributing to the detrimental effects of hypercapnia on the alveolar epithelium, the airways and immune system.

## Graphical abstract

CO_2_ is a signaling molecule that disrupts alveolar fluid clearance and ion transport, impairs alveolar repair, and promotes mitochondrial and endoplasmic reticulum dysfunction. It suppresses innate immunity, host defense, and inflammation, while increasing airway contractility.

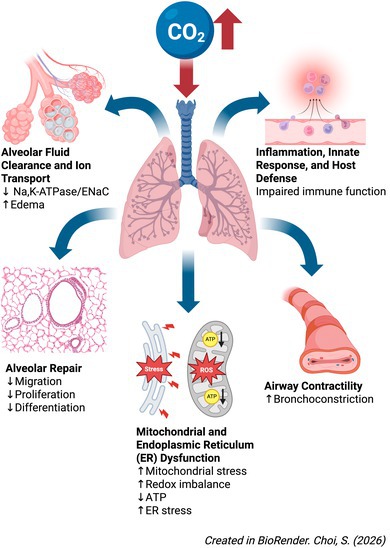

## 1 Introduction

Hypercapnia, defined as an arterial CO_2_ tension (paCO_2_) exceeding 45 mmHg, has pathophysiological effects on cells and organisms. In clinical settings such as acute respiratory distress syndrome (ARDS) and status asthmaticus, elevated CO_2_ has been tolerated as a permissive hypercapnia strategy, which allows moderate hypercapnia to minimize ventilator‐induced lung injury (VILI) by lowering tidal volumes and plateau pressures (Feihl and Perret 1994; Kavanagh and Laffey 2006; Bellani et al. 2016). Early experimental studies suggested that mild CO_2_ elevations attenuated inflammation and oxidative stress, supporting the initial clinical practice adoption (Feihl and Perret 1994; Laffey and Kavanagh 1999; Laffey et al. 2000, 2004). However, more recent studies described the adverse effects of hypercapnia, challenging the paradigm of elevated CO_2_ levels as inert or protective. In a study comparing low versus conventional tidal volumes, higher mortality was associated with patients developing hypercapnia and acidosis (Briva et al. 2007; Vadász et al. 2008; Tiruvoipati et al. 2017). Additional studies reported that acidosis or hypercapnic acidosis were independently associated with intensive care unit (ICU) mortality and more complications during mechanical ventilation in patients with ARDS (Nin et al. 2018). Patients with respiratory disorders such as chronic obstructive pulmonary disease (COPD), asthma, ARDS, pneumonia, pulmonary fibrosis, cystic fibrosis, bronchiectasis, obstructive sleep apnea (OSA), and bronchopulmonary dysplasia can develop hypercapnia (Belkin et al. 2006; Köhnlein et al. 2014; Masa et al. 2015; Piper 2015; Nin et al. 2017). In these settings, paCO_2_ commonly reaches 50–70 mmHg in patients with chronic COPD and may exceed 150 mmHg in severe status asthmaticus during mechanical ventilation (Mutlu et al. 2002; Antro et al. 2005). Importantly, it has been shown that hypercapnia is an independent risk factor for mortality in adult patients with community‐acquired pneumonia (Laserna et al. 2012), children with adenoviral pneumonia (Murtagh et al. 2009), patients with cystic fibrosis awaiting lung transplantation (Belkin et al. 2006) and obesity hypoventilation syndrome (OHS) (Piper 2015).

The biological effects of hypercapnia are time and context dependent. Acute elevations can transiently suppress inflammation and modulate airway contractility, whereas chronic hypercapnia promotes epithelial dysfunction, immune suppression, muscle atrophy, and cellular stress (Jaitovich et al. 2015; Shigemura et al. 2017; Casalino‐Matsuda et al. 2018; Dada et al. 2023; Vadász et al. 2025). The differences between acute versus chronic and the diversity of signaling pathways activated reflect the complexity of elevated CO_2_ effects. This review focuses on mechanistic insights of elevated CO_2_ induced signaling, highlighting pathways such as adenosine monophosphate activated protein kinase (AMPK) activation, Wnt/β‐catenin, NF‐κB regulation, soluble adenylyl cyclase (sAC) signaling, and endoplasmic reticulum (ER) stress that generate tissue‐specific responses regulating ion transport, repair and immunity in the lung.

### 1.1 Alveolar Fluid Clearance and Ion Transport

**FIGURE 1:**
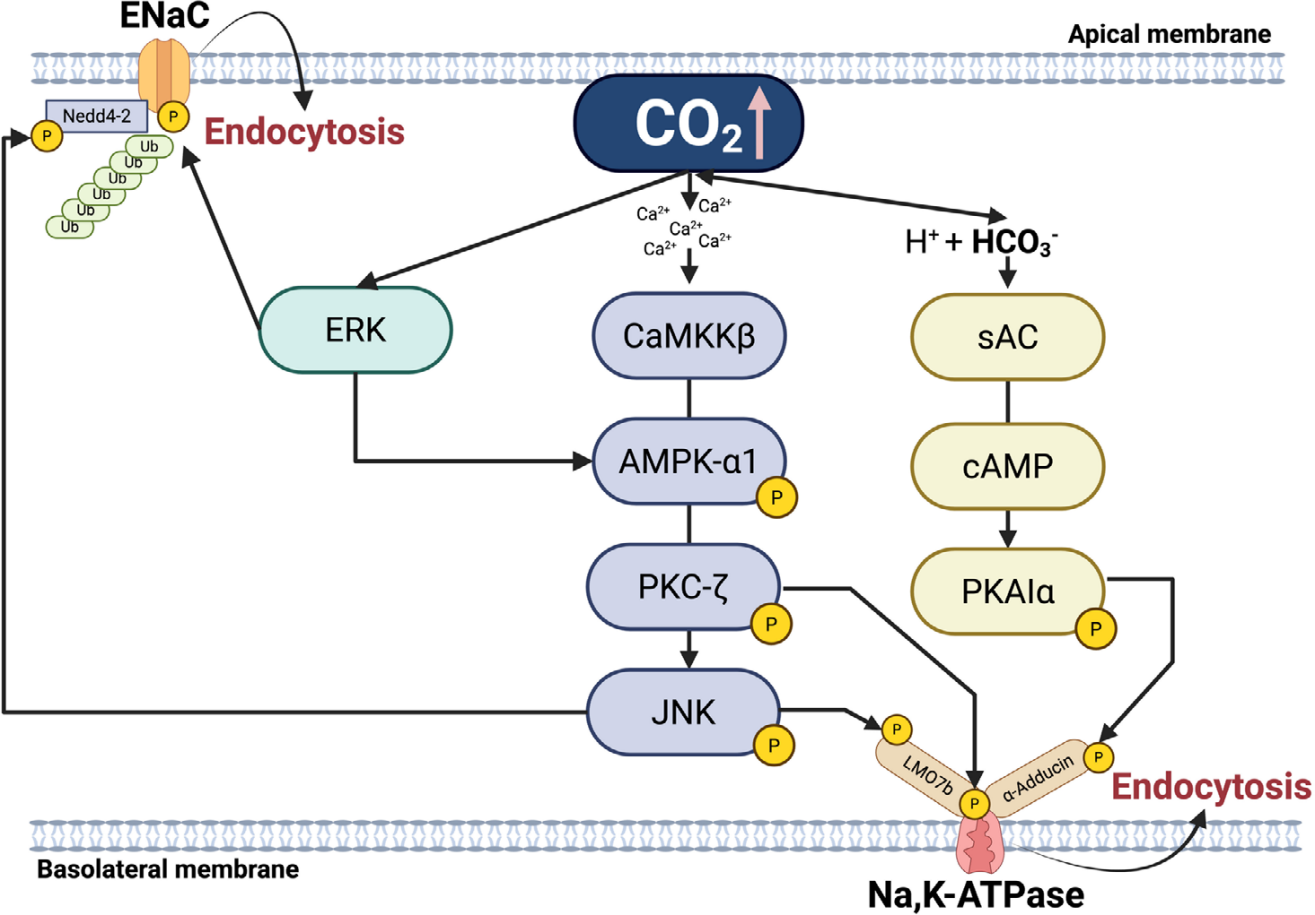
Mechanism of hypercapnia‐induced endocytosis of Na, K‐ATPase and ENaC in alveolar epithelial cells. Created in BioRender Choi, S. (2026).

In patients with ARDS, the alveoli are flooded with edema from leaky capillaries, which interferes with normal gas exchange across the alveolo‐capillary barrier, resulting in impaired oxygenation and CO2 elevation. The alveolar epithelium is composed of cuboidal alveolar type 2 (AT2) cells that secrete surfactant and serve as the stem cell of the alveolar epithelium and squamous alveolar type 1 (AT1) cells, where gas exchange occurs (Barkauskas et al. 2013). Alveolar epithelial fluid clearance (AFC) is essential to remove excess fluid from the alveoli and ensure efficient gas exchange (Sznajder 2001; Dada and Sznajder 2003). This process depends on vectorial sodium transport across the alveolar epithelium, where sodium enters the epithelial sodium channels (ENaC) at the apical membrane and is extruded via the Na,K‐ATPase at the basolateral surface (Sznajder et al. 2002) (Figure 1). The Na,K‐ATPase is composed of a large transmembrane α‐catalytic subunit and a glycosylated regulatory β‐subunit (Blanco and Mercer 1998; Vagin et al. 2012), while ENaC consists of a heterotrimeric structure (α, β, and γ subunits) that are necessary for trafficking and that arrange to form a central ion pore (Eaton et al. 2009, 2010). In AT2 cells, elevated CO_2_ levels increase intracellular Ca^2+^ concentration, which activates Ca^2+^/calmodulin‐dependent protein Kinase Kinase β (CaMKKβ), an upstream activator of AMPK (Vadász et al. 2008). Activated AMPK phosphorylates and stimulates Protein Kinase C‐ζ (PKC‐ζ), which in turn phosphorylates the Na,K‐ATPase α‐subunit, promoting its endocytosis from the plasma membrane, resulting in inhibition of Na,K‐ATPase activity and thus fluid clearance (Vadász et al. 2008) (Figure 1). As a metabolic sensor, AMPK activates pathways that are energy‐producing while inhibiting those that are energy consuming (Hardie et al. 2012). Given that in alveolar epithelial cells, the Na,K‐ATPase consumes ~40% of total resting energy to maintain the electrochemical gradients across the plasma membrane (Comellas et al. 2006), Na,K‐ATPase endocytosis may be a double‐edged sword because while it promotes cell survival under stress conditions, it does it at the cost of fluid accumulation which may result in hypoxemia and hypercapnia.

At the apical surface, elevated CO_2_ causes impaired sodium uptake by inhibiting ENaC activity. Transient activation of Extracellular Signal Regulated Kinase (ERK) phosphorylates the ENaC β‐subunit, enhancing its association with the ubiquitin ligase Nedd4‐2, which mediates ENaC ubiquitination triggering the endocytosis of the complex phosphorylated by JNK (Zhou et al. 2007; Eaton et al. 2010; Gwoździńska et al. 2017) (Figure 1).

High CO_2_ not only activates ERK‐ Mitogen‐activated protein kinase (MAPK), but it also activates c‐Jun N‐terminal kinase (JNK)‐MAPK. While the activation of ERK occurs in seconds and ERK acts upstream of AMPK (Welch et al. 2010), JNK is activated after 5–10 min and acts downstream of AMPK (Vadász et al. 2012). Activation of MAPKs leads to the formation of signaling complexes that include the active kinase and scaffolding proteins whose functions are to regulate the interaction among MAPKs' binding partners (Pearson et al. 2001; Bogoyevitch and Kobe 2006). A screening of such binding partners identified LIM domain‐only 7b (LMO7b), an actin binding protein that regulates protein–protein interactions stabilizing the cortical cytoskeleton (Ooshio et al. 2004), as a JNK substrate (Dada et al. 2015). CO_2_ induced LMO7b phosphorylation weakens the interactions between the Na,K‐ATPase and actin cytoskeleton, facilitating endocytosis of Na,K‐ATPase from the plasma membrane (Dada et al. 2015) (Figure 1). Importantly, this study also provided evidence that exposure to hypercapnia increases the interaction between LMO7b and several proteins involved in clathrin dependent endocytosis, and that LMO7b phosphorylation directs the formation of these vesicles and the recruitment of proteins to the vesicle. Elevated CO_2_ also promotes Na,K‐ATPase endocytosis by sAC activation, a bicarbonate sensitive enzyme that acts as an intracellular CO_2_/HCO_3_^−^ sensor (Buck et al. 1999). sAC generates localized cyclic adenosine monophosphate (cAMP) microdomains distinct from those produced by G‐protein coupled transmembrane adenylyl cyclase (Lecuona et al. 2013). Thus, the sAC dependent activation of endocytosis by CO_2_ contrasts with the well‐known increase by β‐receptor agonist/cAMP protein kinase A (PKA) in the number of Na,K‐ATPase molecules (Bertorello et al. 1999; Lecuona et al. 2009). The selective activation of PKAIα by hypercapnia leads to α‐adducin and other actin‐binding proteins' phosphorylation, inducing actin cytoskeleton reorganization (Lecuona et al. 2013) (Figure 1). As in the case of LMO7b phosphorylation, this remodeling destabilizes Na,K‐ATPase at the plasma membrane and the cytoskeletal anchorage, promoting endocytosis and causing impaired fluid clearance (Lecuona et al. 2013). Taken together, these studies contribute to the concept that elevated CO_2_ acts as a signaling molecule capable of activating pathways which lead to phosphorylation or ubiquitination of specific subunits and scaffolding proteins, thus promoting the endocytosis and subsequent degradation of ion transport proteins.

### 1.2 Alveolar Repair

**FIGURE 2:**
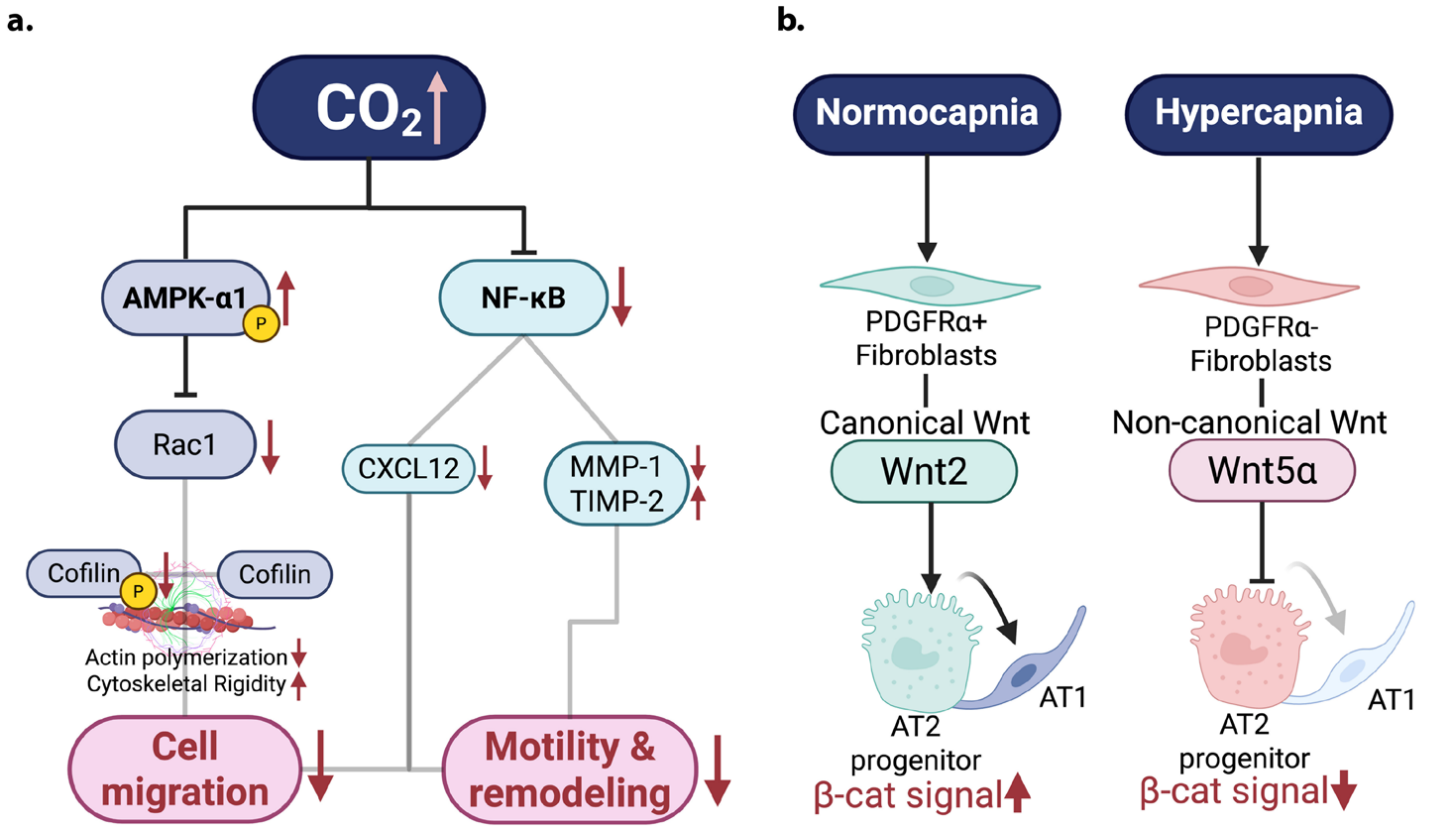
(a) Impaired mechanism of alveolar epithelial repair under hypercapnia in alveolar epithelial cells (b) Schematic of hypercapnia induced disruption of β‐catenin signaling through altered fibroblast Wnt profiles affecting AT2 to AT1 differentiation. Created in BioRender Choi, S. (2026).

Effective epithelial repair is critical for patients to survive lung injury and requires (1) rapid resealing of plasma membranes after injury, (2) directed migration of epithelial cells to restore epithelial continuity, and (3) proliferative renewal and differentiation of AT2 cells into AT1 cells, to reestablish the gas exchange surface area (Barkauskas et al. 2013; Weng et al. 2022; Dada et al. 2023). Hypercapnia contributes to delayed repair by inhibiting NF‐κB dependent transcription of pro‐repair mediators, including cytokines and matrix metalloproteinases expressed by alveolar epithelial cells and macrophages, that normally facilitate motility and extracellular matrix remodeling (O'Toole et al. 2009) (Figure 2a). It has been shown that AT2 migration is dependent on Rac1 dependent cofilin phosphorylation, which is inhibited by hypercapnia (Steffen et al. 2013). CO_2_ dependent AMPK activation diminishes Rac1 and cofilin activity, blunting actin polymerization and lamellipodia formation. The resulting cytoskeletal rigidity restricts cell migration (O'Toole et al. 2009). However, while loss of AMPK rescues Rac1 inhibition it does not rescue epithelial cell migration, indicating that just as in the case of fluid clearance, hypercapnia effects on cell migration are complex and involve multiple signaling cascades. Hypercapnia also downregulates CXCL12, a chemokine essential for epithelial migration and repair, through non‐canonical NF‐κB signaling (Bharat et al. 2020). CXCL12 acts as a key chemoattractant for epithelial and mesenchymal cells (Shigemura et al. 2020), and its inhibition has been linked to poor outcomes in patients with post operative lung injury, where low pleural CXCL12 levels result in delayed healing (Kanter et al. 2015) (Figure 2a). Whereas exogenous CXCL12 supplementation rescues epithelial migration and accelerates repair under hypercapnic conditions, suggesting therapeutic potential (Bharat et al. 2020).

To re‐establish an intact epithelial barrier and recover from lung injury AT2 cells need to proliferate and differentiate into AT1 cells (Hogan et al. 2014). Hypercapnia has been reported to impair AT2 progenitor function (Dada et al. 2023). Alveolar repair depends, among other things, on paracrine signaling between AT2 cells and fibroblasts, mediated largely by canonical Wnt/β‐catenin signaling (Flozak et al. 2010). Wnts are signal molecules that regulate cell fate migration and polarity (Hu et al. 2021). They can be divided into canonical β‐catenin dependent or non‐canonical (or β‐catenin independent) (Baarsma and Königshoff 2017). Canonical Wnt/β‐catenin signals maintain AT2 stemness and their proliferative capacity (Desai et al. 2014; Frank et al. 2016; Nabhan et al. 2018). Hypercapnia has adverse consequences on AT2 progenitor capacity by shifting the Wnt pro‐proliferative signals in the niche surrounding AT2 cells to non‐canonical signals (Dada et al. 2023) (Figure 2b). This defect may be further amplified by CO_2_ induced mitochondrial dysfunction, which impairs ATP generation and biosynthetic capacity required for AT2 proliferation (Vohwinkel et al. 2011). Evidence from 3D organoids and in vivo models demonstrates that sustained hypercapnia reduces AT2 proliferation and lineage progression, whereas canonical Wnt supplementation or β‐catenin activation restores regenerative capacity (Dada et al. 2023).

In summary, sustained hypercapnia prevents the repair of the alveolar epithelial barrier, impairs cell migration, and switches the signals that surround AT2 stem cells towards non‐proliferative signals. Together, these signals lead to increased permeability and alveolar edema resulting in ventilator dependency and increased mortality in patients with acute lung injury.

### 1.3 Mitochondrial and Endoplasmic Reticulum (ER) Dysfunction

We have previously described that in cells exposed to high CO2 levels, cellular proliferation was decreased by a mechanism involving the microRNA‐183 (miR‐183) dependent downregulation of mitochondrial NADP^+^ dependent isocitrate dehydrogenase 2 (IDH2) (Vohwinkel et al. 2011). IDH2 converts isocitrate to alpha ketoglutarate in the tricarboxylic acid cycle (TCA) (Chandel 2021). By generating NADH/NADPH, the TCA supplies electrons to the electron transport chain (ETC). Thus, downregulation of IDH2 by high CO_2_ levels inhibits the regeneration of NAD/NAPD, reduces electron flow, affecting ATP production and potentially increases the production of reactive oxide species (ROS) (Park et al. 2017; Murari et al. 2022).

In parallel, hypercapnia increases CO_2_/bicarbonate (HCO_3_^−^) and chemically redirects ROS/reactive nitrogen species (RNS) into peroxymonocarbonate (HCO_4_^−^) (from hydrogen peroxide, H_2_O_2_) and the carbonate radical (CO_3_•^−^) (from peroxynitrite), accelerating thiol oxidation and causing radical injury to mitochondrial targets, which reinforces ETC dysfunction, ROS production, and redox/energetic failure (Augusto and Truzzi 2021).

This decrease in the production of energy by the mitochondria propagates to the ER, as ER proteostasis depends on NADPH fed glutathione/thioredoxin (GSH/Trx) reducing power for disulfide reduction/isomerization (Ellgaard et al. 2018) and on ATP to power chaperone cycles and sarco/endoplasmic reticulum Ca^2+^ ATPase (SERCA) dependent Ca^2+^ sequestration, which maintains luminal Ca^2+^ required for efficient folding (Kryvenko and Vadász 2021). Consequently, NADPH depletion together with ATP‐limited SERCA activity disrupts ER redox control and Ca^2+^ homeostasis, impairing folding and triggering the unfolded protein response (UPR) and ER stress; additionally, hypercapnia can directly perturb ER proteostasis through lysine carbamylation, which interferes with chaperone binding and nascent peptide folding (Vadász et al. 2025). These changes impair ER quality control and interfere with the maturation of essential proteins such as the Na,K‐ATPase β‐subunit. Under hypercapnic conditions, the altered oxidative environment in the ER causes the β‐subunit to be carbonylated and retained in the rough ER, preventing co‐translational assembly with the catalytic α‐subunit, a process normally coordinated with N‐linked glycosylation and trafficking to the Golgi (Kryvenko, Vagin, et al. 2021). This retention impairs glycosylation, yielding immature, nonfunctional Na,K‐ATPase complexes, thus impairing fluid clearance and barrier integrity.

The UPR has three signaling branches: Inositol requiring protein 1 (IRE1), activating transcription factor 6 (ATF6), and protein kinase RNA (PKR) like ER kinase (PERK) (Kryvenko and Vadász 2021). Persistent ER stress and mitochondrial dysfunction cause the phosphorylation of IRE1α. It has been reported that TNF receptor associated factor 2 (TRAF2), a novel E3‐ligase involved in the ubiquitination of the Na,K‐ATPase β‐subunit is a partner of IRE1α, suggesting the possibility that CO_2_‐induced ubiquitination may regulate the retention of the Na,K‐ATPase β‐subunit (Gabrielli et al. 2021).

Elevated CO_2_ might compromise ER homeostasis through several pathways including JNK and ERK1/2 activation, AMPK engagement, and induction of Bcl‐2 family proteins, HSP70, and caspase‐7 (Shigemura et al. 2018; Kryvenko and Vadász 2021). While these pathways initially promote adaptation and proteostasis restoration, prolonged hypercapnia overwhelms cellular resilience, resulting in apoptosis, impaired epithelial repair, and tissue remodeling (Kryvenko, Wessendorf, et al. 2021). Taken together, the effects of elevated CO_2_ on ER stress, UPR and ERAD and the molecular processes responsible for ER dysfunction illustrate the complexity of the signaling pathways elicited by hypercapnia in lung cells. These signals may be adaptive during acute exposure to help the cells deal with an overload of misfolded protein but become maladaptive with chronic hypercapnic exposure, causing cell dysfunction.

### 1.4 Inflammation, Innate Response, and Host Defense

Multiple reports have described that hypercapnia adversely affects innate immunity and host defense (Helenius et al. 2009; Gates et al. 2013; Restrepo et al. 2018; Love and Proud 2022; Chen et al. 2025). Patients with obstructive lung diseases and hypercapnia, such as severe COPD, are at a higher risk of pulmonary infections, including viral pneumonia, secondary bacterial pneumonia, and community acquired pneumonia (Sin et al. 2005; Sethi and Murphy 2008; Restrepo et al. 2018; Love and Proud 2022). Hypercapnia impairs NF‐κB dependent signals in macrophages, thus reducing the expression of cytokines such as TNF, IL‐1β, and IL‐6 (Wang et al. 2010), and impairing phagocytosis and autophagy (Casalino‐Matsuda et al. 2020). Mice exposed to elevated CO_2_ before or after infection with *Pseudomonas aeruginosa* exhibit decreased early cytokine release, defective neutrophil killing, increased pulmonary bacterial burden, dissemination to extrapulmonary organs, and higher mortality (Gates et al. 2013). Mechanistically, in addition to suppressing NF‐κB driven responses, elevated CO_2_ activates heat shock factor 1 (HSF1) (Lu et al. 2018) and induces Bcl‐2 and Bcl‐xL, two anti‐apoptotic proteins that inhibit autophagy mediated bacterial killing (Casalino‐Matsuda et al. 2015; Lu et al. 2018). Taken together, these observations suggest that the convergence of these signaling pathways impairs alveolar macrophage function and limits neutrophil mediated pathogen clearance, resulting in hypercapnia mediated immunosuppression.

Regarding viral infections, elevated CO_2_ inhibits the expression of antiviral genes, IFN‐β antiviral response and increases viral replication in macrophages and human bronchial epithelial cells infected with influenza A virus (Casalino‐Matsuda et al. 2020; Chen et al. 2025). These effects are mediated by hypercapnia induced activation of Akt signaling, which suppresses interferon stimulated gene expression (Casalino‐Matsuda et al. 2020; Chen et al. 2025). Furthermore, recent evidence suggests that elevated CO_2_ may enhance susceptibility to SARS‐CoV‐2 infection. Hypercapnia upregulates angiotensin converting enzyme 2 (ACE2) expression and promotes viral uptake by increasing cholesterol synthesis and lipid raft accumulation in bronchial epithelial cells, facilitating viral entry (Chen et al. 2023). The effects of hypercapnia on host defense and innate immunity appear to be evolutionarily conserved, as in *Drosophila*, the zinc finger transcription factor ZFH2 mediates CO_2_ induced suppression of antimicrobial peptide genes, while its mammalian ortholog ZFHX3 regulates hypercapnia dependent inhibition of antiviral genes in myeloid cells (Helenius et al. 2016; Casalino‐Matsuda et al. 2024).

Despite its detrimental effects on antimicrobial defense, hypercapnia can exert protective effects in sterile inflammatory conditions. In experimental models of acute lung injury, ischemia and reperfusion, high tidal volume ventilation, and organ transplantation, elevated CO_2_ reduces tissue inflammation by attenuating neutrophil and cytotoxic T‐cell infiltration and enhancing regulatory T‐cell activity (Xie et al. 2024). This immunosuppressive shift limits collateral tissue damage in noninfectious injury, suggesting that hypercapnia modulates immunity in a context‐dependent, dual manner. These findings suggest that elevated CO_2_ sensing as a regulatory mechanism of immune homeostasis but can become maladaptive if hypercapnia is sustained.

### 1.5 Hypercapnia Effects on Airway Contractility

Hypercapnia exerts dual effects on airway tone, with short term CO_2_ elevations often producing bronchodilation, while chronic exposure promotes bronchoconstriction and remodeling. During acute hypercapnia, relaxation of contracted airways can occur, an effect that is epithelium dependent and mediated by local signaling molecules such as substance P (El Mays et al. 2011). Also, the accompanying respiratory acidosis reduces Ca^2+^ influx through voltage dependent calcium channels, lowering intracellular Ca^2+^ and thereby decreasing airway smooth muscle (ASM) contractility (Yamakage et al. 1995). These rapid, reversible effects underscore the context specificity of elevated CO_2_ sensing and highlight intracellular Ca^2+^ as a modulator of gaseous signaling pathways that coordinate ASM responses similarly to those driven by hypoxia and nitric oxide.

In contrast, prolonged hypercapnia is associated with airway hyperreactivity, bronchoconstriction, and structural remodeling, particularly in conditions such as COPD and obesity hypoventilation syndrome (Piper and Grunstein 2011; Mathews et al. 2020).

**FIGURE 3:**
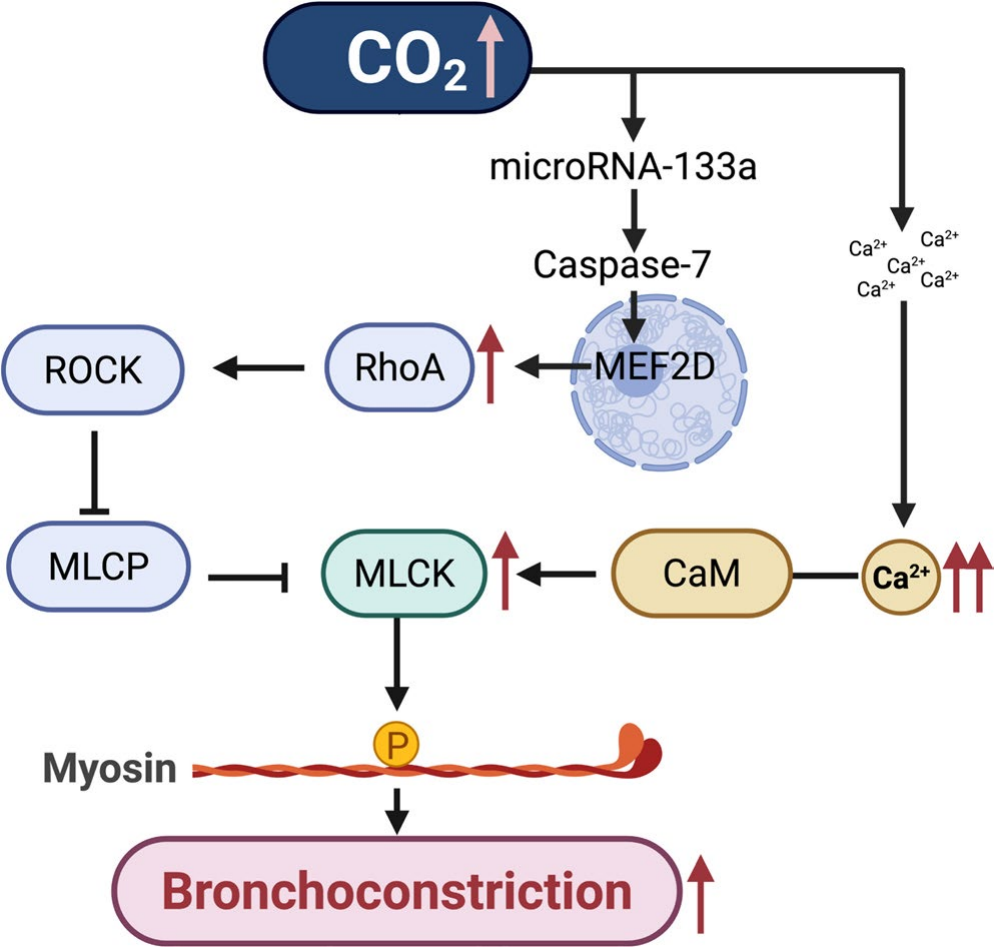
Mechanism of hypercapnia induced bronchoconstriction in airway smooth muscle. Created in BioRender Choi, S. (2026).

At the cellular level, chronic hypercapnia elevates intracellular Ca^2+^, activating Ca^2+^/calpain signaling (Shigemura et al. 2018) (Figure 3). This pathway engages caspase‐7 mediated cleavage of MEF2D, downregulation of microRNA‐133a, and subsequent upregulation of RhoA and myosin light chain phosphorylation, resulting in enhanced contractility (Shigemura et al. 2018) (Figure 3). These signaling events can contribute to airway remodeling with increased deposition of extracellular matrix components such as collagen and laminin.

## 2 Conclusions

Although elevated CO_2_ can transiently activate adaptive stress pathways, increasing evidence suggests that hypercapnia can be harmful. Increased CO_2_ can be sensed by lung cells, in a pH‐independent way, initiating signaling cascades that impair homeostatic lung functions such as fluid reabsorption and host defense, while simultaneously inhibiting cell proliferation and repair after injury. Recognizing elevated CO_2_ as a pathogenic regulator underscores the need to reevaluate permissive hypercapnia and develop strategies that limit CO_2_ elevation or inhibit its downstream signaling.

## Author Contributions

S.C., L.A.D., and J.I.S. drafted, edited, and approved the final version of the manuscript. S.C. made figures.

## Funding

This work was supported by grant NHLBI‐RO1 HL173987.

## Ethics Statement

The authors have nothing to report.

## Conflicts of Interest

The authors declare no conflicts of interest.

## Data Availability

Data sharing not applicable to this article as no datasets were generated or analyzed during the current study.
